# Effects of varying anteroposterior craniodentofacial morphologies on three-dimensional smile variables

**DOI:** 10.1016/j.jds.2025.06.009

**Published:** 2025-06-28

**Authors:** Patcharamas Banditsaowapak, Johnson Hsin-Chung Cheng

**Affiliations:** a Dental Department, Sukumvit Hospital, Bangkok, Thailand; b School of Dentistry, College of Oral Medicine, Taipei Medical University, Taipei, Taiwan; c Orthodontic Division, Department of Dentistry, Taipei Medical University Hospital, Taipei, Taiwan

**Keywords:** Craniofacial biology/genetics, Digital imaging/radiology, Dimensional change, Orthodontic(s), Occlusion

## Abstract

**Background/purpose:**

Research regarding clinical facial assessment has increasingly shifted toward three-dimensional (3D) methods. This cross-sectional study examined the associations between 3D smile variables and two anteroposterior craniodentofacial morphologies (overjet [OJ] and point-A-nasion-point-B angle [ANB]) and quantified facial soft tissue displacement during the transition from rest to posed smiles.

**Materials and methods:**

This study included 119 participants aged 18–30 years. They were divided into three OJ groups (0–4 mm, >4 mm, and <0 mm) and three ANB groups (0°–4°, >4°, and <0°). 3D facial images were taken at rest and during smiling. Subsequently, landmark positions were analyzed. Linear, angular, and proportional measurements were obtained, and landmark displacements were measured.

**Results:**

Of the 257 3D soft tissue variables considered, 41 differed significantly among the 3 OJ groups, and 46 differed significantly among the 3 ANB groups during smiling. The intercommissural width measured during smiling in ANB group 1 was more significant than that in the other two groups. Labiomental angles were larger in ANB group 3 at rest and during smiling, whereas the angle at rest was smaller in OJ group 2. Lower lip movements in OJ group 3 and ANB group 3 were more restricted than those in groups 1 and 2.

**Conclusion:**

OJ and ANB primarily affect soft tissue landmarks during smiling. A large OJ may lead to a deep labiomental sulcus, whereas a negative ANB may result in a flattened sulcus. Reverse OJ and Class III skeletal malocclusion affect the lower lip by restricting its movement.

## Introduction

Several factors influence smile aesthetics, including the maxillary–mandibular skeletal relationship, anterior tooth position, upper lip height and length, age, ethnicity, and sex.^1^, ^2^, ^3^, ^4^, ^5^ Numerous studies have examined the association between smiles and skeletal patterns and have described the importance of evaluating both hard and soft tissues for creating an aesthetically pleasing smile.^6^, ^7^, ^8^, ^9^ However, research evaluating the relationships between smiles and varying craniodentofacial morphologies have mostly used two-dimensional (2D) methods.^6^^,^^10^, ^11^, ^12^, ^13^ Researchers have increasingly been applying three-dimensional (3D) methods for clinical facial assessment that involve optical imaging techniques such as stereophotogrammetry and structured light scanning.^14^, ^15^, ^16^, ^17^ These noncontact and noninvasive imaging techniques provide higher reliability, accuracy, and speed than 2D techniques do.^18^^,^^19^

Numerous studies have used 3D imaging techniques to demonstrate the influence of various vertical skeletal patterns on the soft tissues involved in a smile.^7^, ^8^, ^9^ Nevertheless, studies have not elucidated the variations in soft tissues involved in smiles across different anteroposterior craniodentofacial morphologies. For example, the overjet (OJ), indicating the anteroposterior relationship between the upper and lower incisors, and the point-A-nasion-point-B angle (ANB), representing the anteroposterior relationship between upper and lower jaws, provide influence on facial esthetics and psychological well-being, particularly in patients with various malocclusions.^5^^,^^20^, ^21^, ^22^ Understanding these morphologies assists orthodontists in diagnosing and planning effective treatment strategies aimed at improving both the function and esthetics of the smile.^23^^,^^24^ Campbell et al.^25^ reported that a greater OJ influenced the magnitude of 3D smile variables; however, they did not investigate the effect of a smaller OJ on other smile variables. Novianty et al.^21^ evaluated the ANB angle of hard tissue in relation to the A’N’B angle of soft tissue. Nouh et al.^6^ evaluated 2D smile characteristics of skeletal Class III compared to Class I. The association between ANB and 3D smiles has not been examined. To address these research gaps and provide unique and accurate soft tissue smile measurements in 3D, the current study compared 3D smile variables across various anteroposterior craniodentofacial morphologies (OJ and ANB), quantified facial soft tissue displacement during the transition from rest to posed smiles, and examined the relationship between 3D smile variables and craniodentofacial morphology in patients with different skeletal malocclusions. The results of this study may assist clinicians in improving treatment planning and ultimately lead to more favorable treatment outcomes in the future.

## Materials and methods

### Study design

This cross-sectional study was performed following the STROBE guidelines^26^ and was approved by the Institutional Review Board of Taipei Medical University (Approval No. N202308043).

### Participants and grouping

The required sample size was calculated using G∗Power version 3.1.9.7 (Heinrich-Heine-Universität Düsseldorf, Düsseldorf, Germany). Based on the mean differences among the OJ groups, this study determined that a sample size of at least 3 per group would achieve a statistical power of 80 % and a significance level of 95 %. Thus, 119 Taiwanese adults aged 18–30 years with full dentition (excluding third molars) were included. Individuals with prior orthodontic treatment; congenital, traumatic, or postoperative facial deformity; or plastic surgery were excluded. All data were collected at the Department of Orthodontics, Taipei Medical University Hospital.

The participants were divided into six groups on the basis of OJ^5^ and the point-A-nasion-point-B angle (ANB)^27^ as follows: OJ groups; 0–4 mm, >4 mm, and <0 mm and ANB groups: 0–4°, >4°, and <0°. OJ and ANB were assessed through lateral cephalometric tracing with Viewbox, version 4.1.0.12 (dHAL, Kifissia, Greece).

### 3D facial image collection and measurement

**Figure 1:**
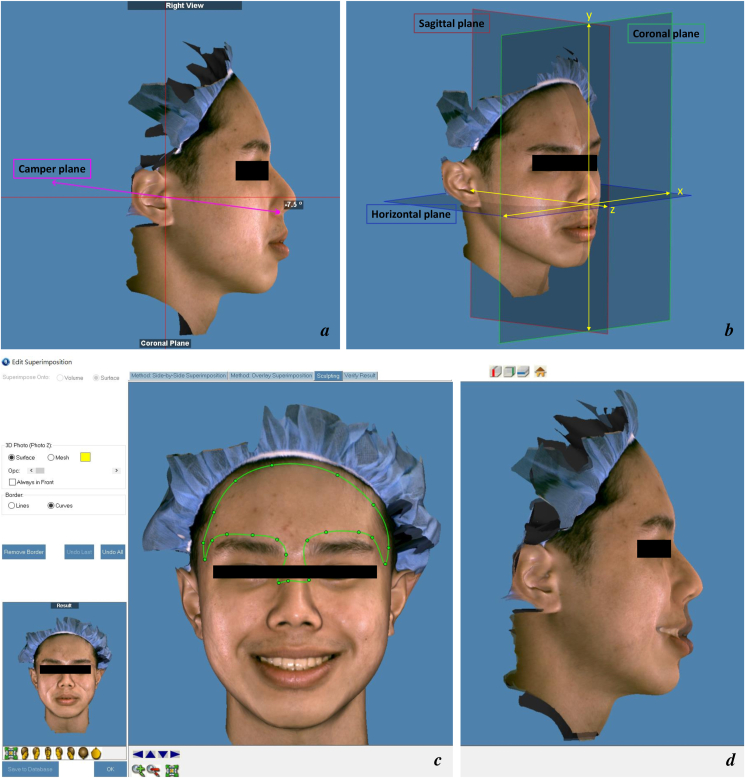
(a) The horizontal plane used in this study was set from the Camper's plane. (b) Soft tissue reference planes were established. (c) 3D resting and smiling images were superimposed on the forehead and nasal root region. (d) A lateral image was obtained of the result of the superimposition.

**Figure 2:**
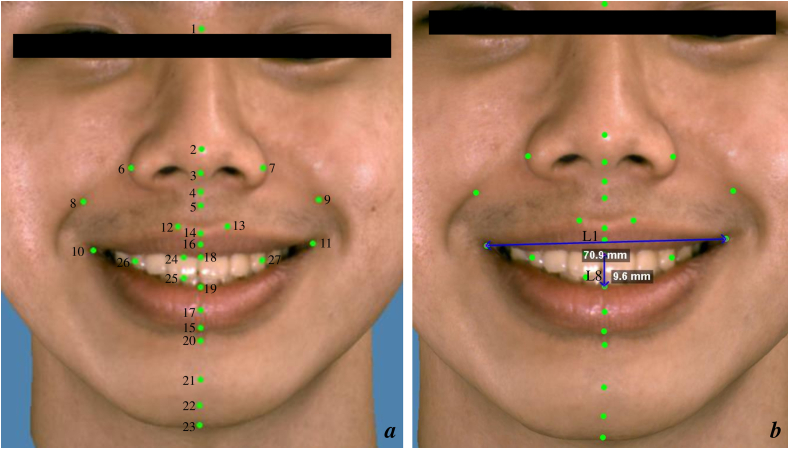
(a) The soft tissue landmarks used in this study were 1. nasion, 2. pronasale, 3. columella, 4. subnasale, 5. subspinale, 6. right alare, 7. left alare, 8. right nasolabial fold, 9. left nasolabial fold, 10. right cheilion, 11. left cheilion, 12. right crista philtre, 13. left crista philtre, 14. labiale superius, 15. labiale inferius, 16. anterior point of the upper lip, 17. anterior point of the lower lip, 18. inferior point of the upper lip, 19. superior point of the lower lip, 20. sublabiale, 21. pogonion, 22. gnathion, 23. menton, 24. right incisor or gum point, 25. right incisor maxilla, 26. right canine point, and 27. left canine point. (b) This figure presents an example of linear measurements (3D line) from a total of 11 measurements; L1: intercommissural width, and L8: interlabial gap.

Soft tissues in the resting and posed smiling positions were analyzed using an Accu3D scanner (Digident Image Technology Co., Ltd., Taichung City, Taiwan [R.O.C.]), a 3D structured light scanner for surface imaging. The resting will be accomplished after complete relaxation for 2 s by training the participants to pronounce the word “Emma”.^28^ The participants were required to say “cheese” while the photo was being taken in order to achieve the posed smile. Before the pictures are taken, the participants must practice smiling three times.^29^ Images were captured at a distance of approximately 45 cm under standard lighting. The reference planes were created using Dolphin imaging software, version 11.9 (Dolphin Imaging and Management Solutions, Chatsworth, CA, USA). To obtain the true horizontal reference, the alare points were rotated 7.5° clockwise^30^ (Fig. 1a). The axial plane was adjusted to pass through the soft tissue subnasale (Sn) point. To create a second reference plane, a sagittal plane was constructed perpendicular to the axial plane. The coronal plane was used as the third reference plane. These reference planes intersected at the Sn point (Fig. 1b). The 3D resting and smiling images were superimposed at the forehead and nasal root points^8^ to enable quantification of facial soft tissue displacement occurring during the transition from rest to a posed smile (Fig. 1c and d). Tracing landmarks (Fig. 2a, Table 1) were added to the 3D images to facilitate the definition and measurement of soft tissue variables.

**Table 1:** Definition of landmarks and measurements in this study.

Landmarks and measurements	Definition
*Landmarks at rest and during smiling*	
1. Nasion (n)	Deepest point of the nasal bridge
2. Pronasale (prn)	Tip of the nose
3. Columella (col)	Point on the lower surface of the nose
4. Subnasale (sn)	Intersection between columella and upper lip
5. Subspinale (SA)	The most posterior midpoint of the philtrum (deepest midline points between the subnasale and labiale superius) or soft tissue point A.
6. Right alare (alr)	Outer points of right nasal alare
7. Left alare (all)	Outer points of left nasal alare
8. Right nasolabial fold (nlfr)	Midpoint of right nasolabial fold
9. Left nasolabial fold (nlfl)	Midpoint of left nasolabial fold
10. Right cheilion (chr)	Right corner points of lips
11. Left cheilion (chl)	Left corner points of lips
12. Right crista philtre (cphr)	Highest points of the right upper vermillion line
13. Left crista philtre (cphl)	Highest points of the left upper vermillion line
14. Labiale superius (ls)	Midpoint of the upper vermillion line
15. Labiale inferius (li)	Midpoint of the lower vermillion line
16. Anterior point of the upper lip (ula)	Most anterior point of the upper lip
17. Anterior point of the lower lip (lla)	Most anterior point of the upper lip
18. Inferior point of the upper lip (uli)	Middle lower point of the upper lip
19. Superior point of the lower lip (lls)	Middle superior point of lower lip
20. Sublabiale (SB)	The most posterior midpoint on the labiomental soft tissue contour that defines soft tissue contour that defines the border between the lower lip and the chin or soft tissue point B.
21. Pogonion (pog)	Most anterior point on the soft tissue contour on the mentum
22. Gnathion (gn)	Midpoint between the most anterior and inferior points on the soft tissue contour of the mentum
23. Menton (me)	Most inferior point below the soft tissue contour of the mentum
*Landmarks visible only during smiling*	
24. Right incisor or gum point (rgu)	Highest point of maxillary right incisors or gingivae observed when smiling
25. Right incisor maxilla (ril)	Lowest point of maxillary right incisors observed when smiling
26. Right canine point (cr)	Midpoint of the buccal surface of the right canine
27. Left canine point (cl)	Midpoint of the buccal surface of the left canine
*3D linear measurements (mm)*	
28. Intercommissural width or mouth width (chr-chl)	Distance between the right and left corners of the mouth
29. Philtrum width (cphr-cphl)	Distance between right and left crista philtri points
30. Upper lip length (sn-uli)	Distance between subnasale and upper lip inferior
31. Upper lip vermillion length (ls-uli)	Distance between labrale superius and inferior point of upper lip
32. Lower lip length (lls-SB)	Distance between superior point of lower lip and labrale inferius
33. Lower lip vermillion length (lls-li)	Distance between superior point of lower lip and sublabiale
34. Total lip vermillion length or mouth height or intervermillion distance (ls-li)	Distance between labiale superius and labiale inferius
35. Interlabial gap (uli-lls)	Distance between inferior point of the upper lip and superior point of the lower lip
36. Nasal arch length (n-prn)	Distance between soft tissue nasion and pronasale
37. Nasal projection (prn-sn)	Distance between soft tissue subnasale and pronasale
38. Nasal width (ra-la)	Distance between right and left alare points
*2D linear measurements (mm)*	
39. Gingival display (uli-rgu)	Right maxillary incisors gingival display length along y-axis
40. Maxillary incisor display (rgu-ril)	Right maxillary incisors display length along y-axis
41. Maxillary intercanine width (cr-cl)	Distance between the right canine point and left canine point along x-axis
42. Right buccal corridors (chr-cr)	Distance between the right canine point and right cheilion along x-axis
43. Left buccal corridors (chl-cl)	Distance between the left canine point and left cheilion along x-axis
*Angular measurements (degree)*	
44. Nasolabial angle (col-sn-ls)	Angle between the columella, subnasale and labiale superius
45. Labiomental angle (li-SB-pog)	Angle between the labiale inferius, sublabiale, and pogonion
46. Upper lip angle (chr-ls-chl)	Angle between the right cheilion, the upper midpoint of the upper lip and the left cheilion
47. Lower lip angle (chr-li-chl)	Angle between the right cheilion, the lowest midpoint of the lower lip and the left cheilion
48. Nasal protrusion angle (alr-prn-all)	Angle between the right alare, pronasale, and the left alare
49. Nasal aspect (n-prn-sn)	Angle between nasion, pronasale point, and subnasale point
*Linear ratio*	
50. Smile index (chr-chl/uli-lls)	Intercommissural width/interlabial gap
51. Intercommissural width to total vermillion length (chr-chl/ls-li)	Intercommissural width/distance between labiale superius and labiale inferius
52. Buccal corridor ratio (cr-cl/chr-chl)	Intercanine width/intercommissural width

Facial movements at rest and during smiling were measured by calculating the displacement of 23 landmarks from the three reference planes on the 3D images. Positive and negative numbers along the x-, y-, and z-axes represented changes in position and direction, where “+” and “−” values indicated positions relative to the right or left, up or down, and forward or backward, respectively. Eleven 3D linear measurements (Fig. 2b), five 2D linear measurements, six angular measurements, and three proportional ratios were obtained. Table 1 presents the definitions of the measurements.

To ensure accuracy, a single examiner obtained measurements twice over a 4-week interval. The reliability of the measurements was tested using intraclass correlation coefficients (ICCs) and Dahlberg's formula.^31^^,^^32^ The ICCs ranged from 0.945 to 0.999 for lateral cephalometric measurements and from 0.740 to 0.998 for soft tissue measurements. The measurement error was 0.31 mm for linear measurements and 0.87° for angular measurements.

### Statistical analysis

The collected data were analyzed using STATA 15.1 (StataCorp LLC, College Station, TX, USA). Skewness and Kurtosis tests were employed to determine whether the data had a normal distribution. Differences were considered significant at *P <* 0.05. Demographic data were evaluated using frequency distributions. ANOVA and Bonferroni post hoc tests were used for data with a normal distribution; specifically, these tests were used to compare 3D landmark positions, linear and angular measurements, linear ratios, and 3D landmark displacements across the OJ and ANB groups. The Kruskal–Wallis test with Dunnett's post hoc test was used to analyze data with a nonnormal distribution. The associations between all 3D smile measurements and craniodentofacial variables were examined using Spearman's correlation coefficient.

## Results

### Demographic data

The average age of the 119 participants (71 women and 48 men) was 22.8 ± 3.2 years. The average OJ and ANB values were 2.0 ± 3.6 mm (3.2 ± 3.2 mm for women and 2.7 ± 3.4 mm for men) and 1.3° ± 3.9° (3.1° ± 3.6° for women and 2.4° ± 3.8° for men), respectively.

### Comparisons of soft tissue variables among three OJ groups and between any two of the three OJ groups

Significant differences were observed in soft tissue variables among the three OJ groups. Significant differences were noted in 14 landmarks on the z-axis at rest (*P* < 0.05). Most of the landmarks were located on the lower third of the face. During smiling, significant differences were identified in nine landmarks on the z-axis among the OJ groups (*P* < 0.05). Most of these landmarks were also located on the lower third of the face.

Significant differences were noted in 10 linear and angular soft tissue measurements among the 3 OJ groups (Table 2). The labiomental angle measured at rest was the smallest in OJ group 2 (*P* = 0.0003), but it did not differ during smiling. At rest and during smiling, the labiomental angle of the upper and lower lips exhibited notable differences among the OJ groups (*P* < 0.05).

**Table 2:** Significant differences in linear and angular soft tissue measurements between the three OJ groups at rest and during smiling, as determined using one-way ANOVA or the Kruskal–Wallis test.

Measurements	Rest					Smile				
	Group 1	Group 2	Group 3	*P*-value (The Kruskal–Wallis test)	*P*-value (ANOVA)	Group 1	Group 2	Group 3	*P*-value (The Kruskal–Wallis test)	*P*-value (ANOVA)
	N = 51	N = 42	N = 26			N = 51	N = 42	N = 26		
	Mean ± SD	Mean ± SD	Mean ± SD			Mean ± SD	Mean ± SD	Mean ± SD		
**3D linear measurements**										
Lower lip vermillion length (lls-li)	12.12 ± 2.47	11.64 ± 1.61	12.48 ± 1.92	0.2029		11.55 ± 2.11	10.94 ± 1.29	12.04 ± 1.98		0.0492^a^
Interlabial gap (uli-lls)	1.76 ± 2.79∗	3.90 ± 4.16†	1.67 ± 2.72		0.0041	10.48 ± 3.55	11.51 ± 3.38	11.10 ± 3.55		0.3584
Nasal projection (prn-sn)	19.26 ± 2.19∗	18.24 ± 1.79	18.42 ± 1.65		0.0309	20.06 ± 2.30	19.32 ± 2.04	19.45 ± 1.83		0.2101
**2D linear measurements**										
Maxillary incisor display (rgu-ril)						6.68 ± 2.64†	7.32 ± 2.44†	4.60 ± 2.84		0.0002
**Angular measurements**										
Labiomental angle (li-SB-pog)	133.42 ± 10.96∗	127.47 ± 10.88†	139.69 ± 14.34		0.0003	133.99 ± 11.73	131.07 ± 8.89	137.16 ± 7.50		0.0515
Upper lip angle (chr-ls-chl)	98.16 ± 6.07	94.46 ± 7.85†	103.99 ± 5.47		<0.0001	99.35 ± 6.87	97.02 ± 6.37†	103.07 ± 7.72		0.0028
Lower lip angle (chr-li-chl)	109.89 ± 6.90†	111.21 ± 9.16†	104.25 ± 7.18		0.0016	95.38 ± 6.57†	96.94 ± 5.91†	88.70 ± 6.30		<0.0001
Nasal protrusion angle (alr-prn-all)	93.26 ± 7.41	96.53 ± 6.58	96.11 ± 5.31		0.0464^a^	98.12 ± 5.70	100.28 ± 5.94	99.84 ± 5.00		0.1592

∗*P* < 0.05, compared with group 2.

†*P* < 0.05, compared with group 3.

a No significant difference between paired comparison with post-hoc (Bonferroni) test.

Significant differences were observed in the movement distances of six landmarks from resting to smiling positions on the x-, y-, and z-axes among the three OJ groups (Table 3). The lateral movement of the inferior point of the upper lip on the x-axis was greater in OJ group 3 than in OJ groups 1 and 2 (*P* = 0.0247), and it exhibited a symmetrical pattern. The backward movement of the inferior point of the upper lip on the z-axis was greater in OJ group 3 than in OJ groups 1 and 2 (*P* = 0.0058). By contrast, the backward movement of the superior point of the lower lip was smaller in OJ group 3 than in OJ groups 1 and 2 (*P* = 0.0298).

**Table 3:** Significant differences in landmark movement distances from resting to smiling positions on the x-, y-, and z-axes between the three OJ groups, as determined using one-way ANOVA or the Kruskal–Wallis test.

Soft tissue landmarks	x-axis					y-axis					z-axis				
	Group 1	Group 2	Group 3	*P*-value (The Kruskal–Wallis test)	*P*-value (ANOVA)	Group 1	Group 2	Group 3	*P*-value (The Kruskal–Wallis test)	*P*-value (ANOVA)	Group 1	Group 2	Group 3	*P*-value (The Kruskal–Wallis test)	*P*-value (ANOVA)
	N = 51	N = 42	N = 26			N = 51	N = 42	N = 26			N = 51	N = 42	N = 26		
	Mean ± SD	Mean ± SD	Mean ± SD			Mean ± SD	Mean ± SD	Mean ± SD			Mean ± SD	Mean ± SD	Mean ± SD		
Pronasale (prn)	0.01 ± 0.17	−0.02 ± 0.26	0.02 ± 0.22	0.5810		−0.15 ± 0.54	0.02 ± 0.72†	−0.40 ± 0.75		0.0370	−1.04 ± 0.69	−0.92 ± 0.62	−0.97 ± 0.63		0.6742
Columella (col)	−0.01 ± 0.17	−0.04 ± 0.18	0.01 ± 0.35	0.7757		−0.32 ± 0.51	−0.04 ± 0.63†	−0.50 ± 0.76		0.0085	−1.91 ± 1.18	−1.49 ± 1.35	−1.69 ± 0.86		0.2358
Left cheilion (chl)	5.73 ± 2.99	5.65 ± 2.56	4.78 ± 2.20		0.3121	6.16 ± 3.18	6.05 ± 2.50	4.48 ± 2.77		0.0411^a^	−11.14 ± 3.80	−11.75 ± 4.40	−11.54 ± 4.01		0.7585
Inferior point of the upper lip (uli)	0.25 ± 0.78	−0.01 ± 0.42†	0.44 ± 0.76	0.0247		4.56 ± 2.47	3.80 ± 2.79	3.84 ± 2.38		0.2998	−5.03 ± 2.38†	−4.55 ± 2.27†	−6.61 ± 3.27		0.0058
Superior point of the lower lip (lls)	0.16 ± 0.49	0.06 ± 0.57	0.31 ± 0.91	0.7580		−3.37 ± 2.93	−3.24 ± 2.84	−4.48 ± 3.73	0.3127		−6.94 ± 2.44†	−6.72 ± 2.98†	−5.22 ± 2.94		0.0298

∗*P* < 0.05, compared with group 2.

†*P* < 0.05, compared with group 3.

a No significant difference between paired comparison with post-hoc (Bonferroni) test.

### Comparisons of soft tissue variables among the three ANB groups and between any two of the three ANB groups

At rest, significant differences were observed in 10 landmarks on the z-axis among the three ANB groups (*P* < 0.001), with the most anterior location being noted in group 3 (*P* < 0.001). Significant differences were noted in the positions of the gnathion and menton landmarks on both the z- and y-axes between ANB groups 2 and 3 (*P* < 0.05). The positions of these two landmarks were higher in ANB group 2 than in ANB groups 1 and 3.

During smiling, significant differences were noted in 14 landmarks among the 3 ANB groups (*P* < 0.005). The right and left cheilions were positioned furthest from the x-axis in ANB group 1, followed by in ANB groups 2 and 3.

Significant differences were noted in 11 linear and angular soft tissue measurements among the 3 ANB groups (Table 4). The intercommissural width measured during smiling was the largest in ANB group 1. This width in ANB group 1 differed significantly from that in group 3 (*P* = 0.0107). The maxillary incisal display measured during smiling was significantly smaller in ANB group 3 than in ANB groups 1 and 2 (*P* = 0.0163). The labiomental angles both at rest and when smiling were significantly larger in ANB group 3 than in ANB groups 1 and 2 (*P* = 0071 and 0.013, respectively). Upper lip angles were also significantly larger in group 3 than in groups 1 and 2 (*P* = 0.0001 for rest and *P* < 0.0001 for smiling). By contrast, lower lip angles were smaller in this group relative to in groups 1 and 2 (*P* = 0.0035 for rest and *P* < 0.0001 for smiling).

**Table 4:** Significant differences in linear and angular soft tissue measurements between the three ANB groups at rest and during smiling, as determined using one-way ANOVA or the Kruskal–Wallis test.

Measurements	Rest					Smile				
	Group 1	Group 2	Group 3	*P*-value (The Kruskal–Wallis test)	*P*-value (ANOVA)	Group 1	Group 2	Group 3	*P*-value (The Kruskal–Wallis test)	*P*-value (ANOVA)
	N = 41	N = 46	N = 32			N = 41	N = 46	N = 32		
	Mean ± SD	Mean ± SD	Mean ± SD			Mean ± SD	Mean ± SD	Mean ± SD		
**3D linear measurements**										
Intercommissural width or mouth width (chr-chl)	48.75 ± 3.77	46.66 ± 5.03	47.17 ± 3.68		0.0703	60.36 ± 6.38†	57.49 ± 5.20	56.53 ± 5.33		0.0107
Lower lip vermillion length (lls-li)	12.38 ± 2.57	11.66 ± 1.62	12.11 ± 1.99	0.4798		12.00 ± 1.98∗	10.75 ± 1.56†	11.72 ± 1.85		0.0041
Interlabial gap (uli-lls)	1.83 ± 2.80	3.51 ± 4.20	1.89 ± 2.73		0.0390^a^	10.47 ± 2.82	11.55 ± 4.00	10.80 ± 3.49		0.3390
**2D linear measurements**										
Maxillary incisor display (rgu-ril)						6.98 ± 2.48†	6.81 ± 2.81†	5.26 ± 2.85		0.0163
Maxillary intercanine width (cr-cl)						39.68 ± 3.33∗	37.50 ± 3.99	37.87 ± 4.30	0.0281	
**Angular measurements**										
Labiomental angle (li-SB-pog)	131.43 ± 12.67†	129.80 ± 10.60†	138.45 ± 13.26		0.0071	131.49 ± 10.54†	132.51 ± 9.58†	138.07 ± 9.31		0.0130
Upper lip angle (chr-ls-chl)	98.85 ± 5.91∗†	93.81 ± 7.43†	103.41 ± 5.45	0.0001		100.46 ± 5.55∗	95.69 ± 6.78†	103.16 ± 7.33		<0.0001
Lower lip angle (chr-li-chl)	109.93 ± 7.10†	111.19 ± 8.79†	105.12 ± 7.42		0.0035	94.71 ± 5.70†	97.56 ± 6.55†	89.73 ± 6.62		<0.0001

∗*P* < 0.05, compared with group 2.

†*P* < 0.05, compared with group 3.

a No significant difference between paired comparison with post-hoc (Bonferroni) test.

Significant differences were observed in seven landmark from resting to smiling on the x-, y-, and z-axes among the three ANB groups (Table 5). The lateral movement of cheilions on the x-axis was the largest in ANB group 1. Significant differences were noted in the movements of the anterior point of the lower lip, inferior point of the upper lip, and superior point of the lower lip on the z-axis (*P* = 0.0001, *P* = 0.0085, and *P* = 0.0006, respectively). In group 2, the backward movement of the anterior point of the lower lip and superior point of the upper lip was the greatest, whereas the backward movement of the inferior point of the upper lip was the least notable.

**Table 5:** Significant differences in landmark movement distances from the resting position to the smiling position along the x-, y-, and z-axes between the three ANB groups, as determined using one-way ANOVA or the Kruskal–Wallis test.

Soft tissue landmarks	x-axis					y-axis					z-axis				
	Group 1	Group 2	Group 3	*P*-value (The Kruskal–Wallis test)	*P*-value (ANOVA)	Group 1	Group 2	Group 3	*P*-value (The Kruskal–Wallis test)	P-value (ANOVA)	Group 1	Group 2	Group 3	*P*-value (The Kruskal–Wallis test)	*P*-value (ANOVA)
	N = 41	N = 46	N = 32			N = 41	N = 46	N = 32			N = 41	N = 46	N = 32		
	Mean ± SD	Mean ± SD	Mean ± SD			Mean ± SD	Mean ± SD	Mean ± SD			Mean ± SD	Mean ± SD	Mean ± SD		
Pronasale (prn)	0.03 ± 0.17	−0.04 ± 0.25	0.02 ± 0.20	0.2129		−0.26 ± 0.67	0.07 ± 0.63	−0.29 ± 0.66		0.0223^a^	−0.99 ± 0.78	−0.97 ± 0.56	−0.99 ± 0.62		0.9867
Columella (col)	−0.02 ± 0.20	−0.02 ± 0.16	0.00 ± 0.31	0.3995		−0.48 ± 0.63∗	0.00 ± 0.55†	−0.35 ± 0.63		0.0008	−1.85 ± 1.25	−1.58 ± 1.27	−1.74 ± 0.98		0.5692
Right nasolabial fold (nlfr)	−3.47 ± 2.33†	−2.94 ± 1.92	−2.16 ± 1.96	0.0239		4.82 ± 2.56	4.42 ± 2.74	3.66 ± 2.67		0.1798	−4.35 ± 1.56	−3.92 ± 2.14	−3.56 ± 2.39		0.2572
Labiale inferius (li)	0.13 ± 0.65	0.14 ± 0.48	0.28 ± 0.65		0.5162	−2.84 ± 2.53	−2.36 ± 3.28	−3.50 ± 4.24	0.5357		−5.06 ± 2.41∗	−6.73 ± 2.65†	−4.64 ± 2.51		0.0007
Anterior point of the lower lip (lla)	0.11 ± 0.59	0.13 ± 0.44	0.29 ± 0.73		0.3601	−3.27 ± 2.60	−2.74 ± 3.25	−3.66 ± 4.09		0.4692	−5.33 ± 2.49∗	−7.18 ± 2.77†	−4.67 ± 2.58		0.0001
Inferior point of the upper lip (uli)	0.05 ± 0.60†	0.15 ± 0.57	0.46 ± 0.87		0.0288	4.76 ± 2.69	3.77 ± 2.54	3.86 ± 2.37		0.1554	−5.13 ± 2.31	−4.48 ± 2.48†	−6.34 ± 2.99		0.0085
Superior point of the lower lip (lls)	0.08 ± 0.61	0.12 ± 0.44	0.31 ± 0.84	0.6785		−3.28 ± 2.02	−3.48 ± 3.39	−4.06 ± 3.79	0.6149		−6.52 ± 2.46	−7.47 ± 2.78†	−5.03 ± 2.73		0.0006

∗*P* < 0.05, compared with group 2.

†*P* < 0.05, compared with group 3.

a No significant difference between paired comparison with post-hoc (Bonferroni) test.

### Correlations between craniodentofacial variables and 3D smile measurements (soft tissue variables)

The correlations between craniodentofacial variables and 3D smile measurements were analyzed using Spearman's correlation. Strong correlations were observed between craniodentofacial variables and soft tissue variables. Strong negative correlations (<−0.7) were observed between ANB and soft tissue landmark positions on the z-axis. A moderate negative correlation was observed between OJ and several soft tissue landmark positions on the z-axis. Furthermore, a moderate positive correlation (0.628) was noted between OJ and ANB.

## Discussion

This study elucidated the anteroposterior craniodentofacial variables influencing soft tissue smile variables, particularly on the z-axis. Our measurements at rest align with the soft tissue cephalometric analysis by Arnett et al.^33^ Their true vertical line and soft tissue landmark projections are comparable to our landmark positions at rest on the z-axis. Unlike studies on Angle Class I malocclusion, this study included participants with various ANB values to better match skeletal features. Cheng et al.^2^ suggested that malocclusion classes correspond to skeletal patterns. The positions of the pronasale, Sn, subspinale, anterior upper and lower lip points, sublabiale, and pogonion at rest on the z-axis align with Arnett et al.^33^ However, the pronasale was positioned more forward and was shorter, while the anterior points of the lips moved further along the z-axis, likely due to ethnic differences.

Regarding linear and angular measurements and soft tissue ratios, the intercommissural width during smiling was greatest in ANB group 1, with significant differences between ANB groups 1 and 3. However, no significant differences were found at rest. The right and left cheilions were furthest on the x-axis in ANB group 1, with significant differences between ANB groups 1 and 3. This suggests Class III malocclusion may restrict mouth width during smiling. The labiomental angle was largest in ANB group 3 and smallest in ANB group 2. At rest, it was smallest in OJ group 2. Arnett and Bergman^34^ found that a smaller mandibular sulcus contour is linked to Class II malocclusion with vertical maxillary deficiency or deep bite, while a larger contour is associated with Class III mandibular protrusion and lower lip tension. Our findings align with their results on anteroposterior dentoskeletal malocclusion.

This study compared the displacements of facial soft tissue landmarks at rest and during smiling between the three OJ groups. The results revealed significant differences in landmark movements between OJ groups 3 and 2 and some significant differences between OJ groups 3 and 1. No significant difference was observed in landmark movements between OJ groups 1 and 2. These findings differ from those reported by Campbell et al.,^25^ who observed greater mean movement in the normal OJ group than in the large OJ group. However, they did not report on the smile movements of participants with reverse OJ. By contrast, in the present study, with the exception of for the superior point of the lower lip, the movements of all landmarks on all three axes were the greatest in OJ group 3. Similar results were obtained for the ANB groups. That is, the backward movement of the labiale inferius, anterior point of the lower lip, and superior point of the lower lip on the z-axis was the lowest in ANB group 3. These results suggest that reverse OJ and Class III skeletal malocclusion restrict the movement of the lower lip.

Regarding the correlations between craniodentofacial variables and 3D soft tissue variables, we observed strong and moderate negative correlations between ANB and soft tissue landmark positions on the z-axis. In addition, we observed a moderate negative correlation between OJ and soft tissue landmark positions on the z-axis. These observations were obtained for the measurements at rest and during smiling. This study confirmed that the degree of OJ and ANB influences soft tissue smile variables. Additionally, the moderate positive correlation between OJ and ANB suggests that the normal, large, and reverse overjet groups may correspond to Class I, Class II, and Class III skeletal morphologies, respectively, aligning with the findings of the 2D study conducted by Cheng and Cheng.^5^

Our research revealed influencing factors in both dental and skeletal components; thus, we could infer which component influences smile measurements. The intercommissural width was influenced by ANB, but not by OJ. Consequently, simply modifying the OJ through purely orthodontic treatment may not affect the intercommissural width. As a result, we could develop an effective treatment plan for traditional orthodontic or orthognathic surgery by addressing the contributing elements to get a more esthetically pleasing smile. However, the generalizability of the study results may be limited. Sex and facial dimensions could be potential confounding factors in our study. In the current study, the small sample size poses a challenge when considering potential confounding factors, as it may also compromise statistical power. This is one of the limitations in our study. However, we recognize that sex is an important factor influencing smile variables. Collecting a larger and more balanced sample to further investigate such as potential sex-related confounding effects, as well as utilizing a generalized Procrustes analysis^35^ to compensate for individual facial dimension differences are needed for future research. The uneven sample sizes across OJ and ANB groups may compromise statistical power and the reliability of group comparisons as one of the study limitations. Enlarging the sample size possibly in future studies would not only enhance the statistical power but also allow for better control of potential confounding factors. This would contribute to more reliable group comparisons and a more comprehensive understanding of the variables influencing the observed outcomes. Additionally, further investigations should be randomized, with an equal number of samples. A comparison of 3D smile variables before and after orthodontic treatment was recommended. Furthermore, we could quantify lip thickness by combining it with 3D intraoral images to investigate additional smile morphology.

In conclusion, this study revealed that of 257 3D soft tissue variables, significant differences were noted in 41 between the 3 OJ groups and in 46 between the 3 ANB groups. A large OJ may cause a deep labiomental sulcus, whereas a negative ANB value (indicating Class III skeletal malocclusion) may result in a flattened sulcus. Additionally, reverse OJ and Class III skeletal malocclusion restrict the movement of the lower lip. Spearman's correlation results revealed that the degree of OJ and ANB influence soft tissue smile variables, particularly landmark positions on the z-axis.

## Declaration of competing interests

The authors have no conflicts of interest relevant to this article.
